# Engaging with adolescents to navigate the Adolescents’ Resilience and Treatment nEeds for Mental health in Indian Slums (ARTEMIS) trial

**DOI:** 10.7189/jogh.12.03084

**Published:** 2022-12-21

**Authors:** Sandhya Kanaka Yatirajula, Sudha Kallakuri, Srilatha Paslawar, Ankita Mukherjee, Naushad Alam Khan, Kamala Kumari, Rajesh Sagar, Graham Thornicroft, Pallab K Maulik

**Affiliations:** 1 The George Institute for Global Health, New Delhi, India; 2 All India Institute of Medical Sciences, New Delhi, India; 3 Centre for Global Mental Health and Centre for Implementation Science, Institute of Psychiatry, Psychology and Neuroscience, King’s College London, London, UK; 4 University of New South Wales, Sydney, Australia

Adolescence causes rapid changes at physical, mental, and social levels rending adolescents vulnerable to stress, depression, and increased risk of self-harm/suicide [1] In India, mental illness is one of the leading causes of death and disability for this age group [2].

Many adolescents with mental disorders worldwide are left untreated and unidentified [3] due to lack of awareness of mental health needs, public stigma and internalised stigma, and few mental health professionals available. The situation is worse for slum-dwelling adolescents who are particularly vulnerable to mental disorders due to greater poverty, unemployment, low parental education, poor living conditions and limited access to mental health care [4]. This makes it essential to develop strategies to directly support and treat adolescents in slums who have mental health conditions.

A popular methodology which is gaining momentum in research and implementation of mental health interventions globally is adolescent-practitioner-researcher collaboration and partnership [5,6]. Such collaborations provide useful insights that inform the development and delivery of programmes with adolescents [7]. Such co-creation focuses on the use of participatory methods in which the target group is actively involved in developing, implementing, and evaluating [8] intervention strategies meant for them. Co-creation results in contextually appropriate tailor-made interventions that have greater ownership [9], which empowers the target group, and leads to increased adherence and effectiveness. It is especially valuable when researchers seek to learn and work with marginalised or high-risk populations [10].

This viewpoint paper outlines the process of co-creation with adolescents as part of the Adolescents’ Resilience and Treatment nEeds for Mental health in Indian Slums (ARTEMIS) project. The reflections, learnings from engagements with adolescents, and the challenges encountered are also discussed.

## ARTEMIS AND ITS ADOLESCENT EXPERT ADVISORY GROUP (AEAG)

ARTEMIS is a cluster randomised control trial being implemented in urban slums of New Delhi and Vijayawada cities in north and south India. ARTEMIS is an intervention that combines an anti-stigma campaign with a technology enabled mental health service delivery model to reduce depression and risk of suicide among adolescents in selected slums in these two cities. It aims to test whether a community-based anti-stigma campaign leads to significant improvements in community behaviours toward adolescents with mental illness, and whether an electronic decision support system can improve the treatment of adolescents at high-risk of depression and/or suicide [11]. A critical component of the ARTEMIS is the establishment of adolescent expert advisory groups (AEAGs) to guide the researchers on all aspects of the project design, development, and implementation. The members of the AEAG are instrumental in providing inputs on the feasibility and acceptability of the materials/strategies of the anti-stigma component of the intervention. AEAGs, comprising of adolescents drawn from the slums, collaborated with researchers to conceptualise and execute an adolescent friendly anti-stigma campaign using strategies that they deemed contextually relevant. The primary aim of establishing the AEAGs is to have a group of adolescent experts drawn from the community to collaborate with project staff to conceptualise and execute an adolescent friendly anti-stigma campaign for their area using strategies that are identified as contextually relevant.

## THE FORMATION OF THE AEAG HAD THREE DISTINCT STAGES

### Creation of Standard Operation Procedures (SOP)

A SOP document was created at the outset to guide researchers on the modalities of engaging with the AEAG. The primary criteria for selection of AEAG members was that they were slum-dwelling adolescents (10 to 19 years old), interested in being part of the AEAG, had the time and ability to articulate and contribute to discussions, or other engagements, and that they or their parents had given informed consent.

It was envisaged that that the AEAG members would participate in structured discussions facilitated by project staff and would provide feedback and inputs for strengthening the intervention and achieving project goals by conceptualising an adolescent friendly anti-stigma campaign that is suitable and relevant in their setting, as well as contribute to the mhealth component of the project.

### Early engagement meetings

**Figure 1:**
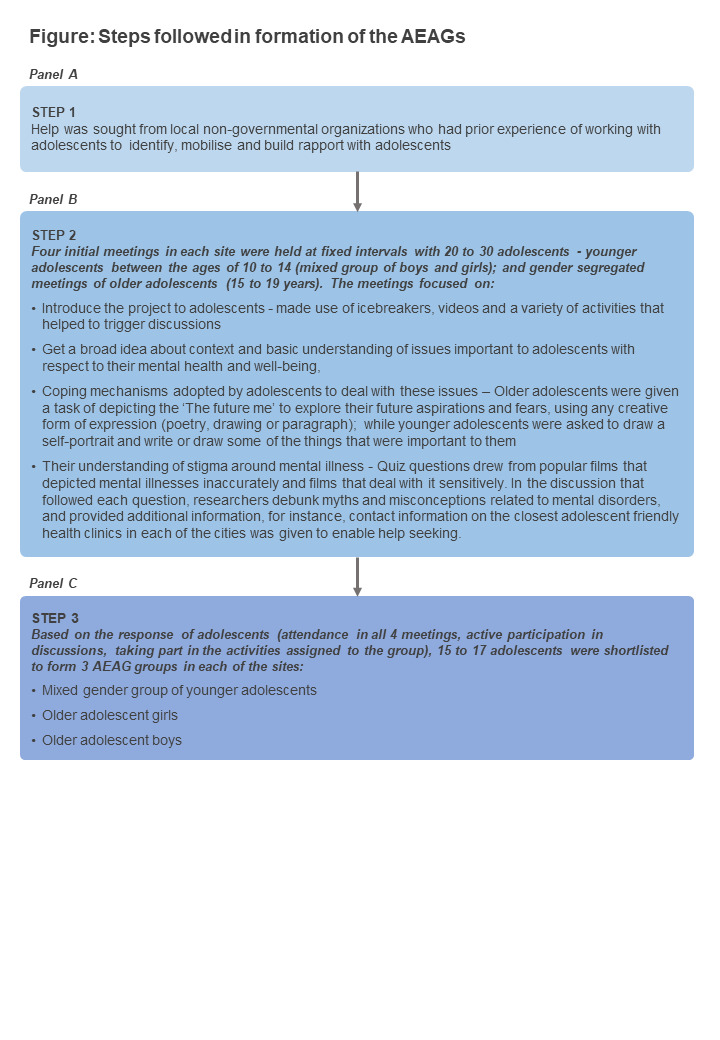
Steps followed in formation of the Adolescent Expert Advisory Group (AEAGs).

A detailed plan was designed to ensure consistency in setting up the AEAG across both sites. Selection of interested volunteers for the AEAGs were done over multiple meetings (**Figure 1**). The plan included information about the content of initial meetings with adolescents, frequency of these initial meetings, different activities to be planned for each meeting and the criteria for selecting the AEAG members. A streamlined selection process was formulated involving multiple initial contact meetings, to find interested volunteers for the AEAG. Recognising that the use of imagery can enable more effective participation [12], the researchers made extensive use of videos and pictures to elicit responses.

### Formation of AEAGs and development of the anti-stigma campaign

Once the AEAGs were formed, meetings were held to explain about ARTEMIS, the role of AEAGs, and the principles that would guide interactions between researchers and adolescents. Subsequent meetings were held to gain insights into AEAGs understanding of common mental conditions such as depression, stress, suicide, alcohol and substance use, and stigma. The AEAG members identified numerous stressors that they faced - academic, parental and peer pressures, gendered restrictions, and future career concerns. The meetings also sourced information from AEAG members on shaping the anti-stigma campaign and identifying the most effective communication methods to use with other adolescents living in similar settings to tackle the issue of stigma associated with mental health. The AEAG identified anti-stigma campaigns as an effective strategy to enhance understanding and support for adolescents with mental disorders in the community. In ARTEMIS, adolescent members of the AEAG have been visualised as co-creators of the anti-stigma campaign, therefore, the research team and project supervisors/project managers (practitioner) first discussed the contents of the campaign materials with the AEAG.

**Figure:**
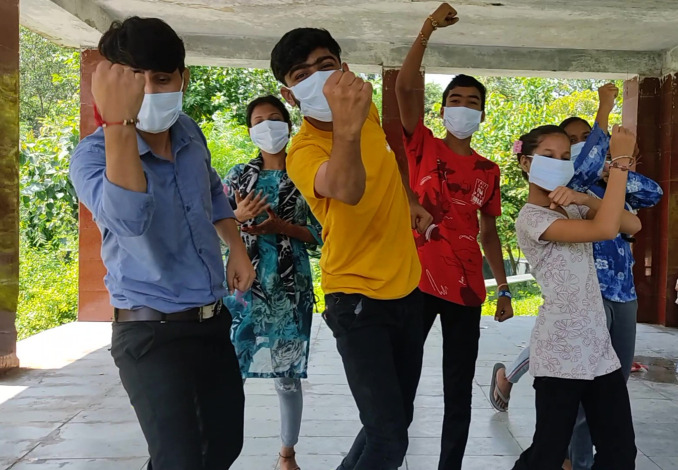
Photo: Adolescent Expert Advisory Group members performing a street play. Source: ARTEMIS Archives. Informed consent was sought from all AEAG members over 18 years old. For adolescents under 18, informed consent was sought from their parents/legal guardians and assent was sought from the adolescents themselves to use photos/videos of AEAG members participating in various activities related to the ARTEMIS project.

Key suggestions made by AEAG were then discussed among the researchers and project supervisors/project managers and were used to develop resource materials and implementation strategies. These resources and strategies were taken back to the AEAG for inputs and suggestions and were refined through repeated iterative processes during regular meetings convene over a 10-month period. During these meetings, AEAG members identified communication methods to improve community and adolescents’ attitudes about mental health of adolescents. The AEAG members wrote plots for street plays which would force people to examine their conservative attitudes and its impact on the mental well-being of adolescents. They also underlined the importance of organising activities especially targeted to creating awareness among parents. Besides parents, the AEAG identified other key stakeholders (other adolescents, older community members, teachers, and tuition centres) towards whom the messages should be directed by holding community meetings, taking out rallies and using IEC materials. They suggested creating short videos starring the adolescents themselves or digital stories that conveyed relevant messages, which could be shared through social media. They also suggested using games to convey the messages and agreed that a magic show that incorporated messages to promote help-seeking and tackle stigma would find favour not only among adolescents but with other community members as well. However, they categorically rejected the use of comic books as most felt that it needed patience to read. As an alternative short audio dramas and animation films were suggested.

## CHALLENGES

This process of AEAG formation was not without challenges and led to key learnings. COVID forced the researchers to engage with adolescents using online strategies whenever restrictions were in place. also, time is a constraint with adolescents being busy with school, tuitions, examinations, and jobs.

## KEY LEARNINGS

Access to younger adolescents and older girls, was possible only after gaining community-level trust which was enabled by local NGOs who had prior experience working with adolescents in the community.

Co-creation ensured that the intervention was culturally and contextually relevant for both sites, thus making it inclusive and potentially more scalable. Co-creation also made it possible to engage adolescents with lived experience of mental disorders. Age played a crucial role in degree of engagement with adolescents. While older adolescents were vested in campaign components and provided the research team with new ideas, they were unable to spare time for more detailed contributions. On the other hand, younger adolescents were able to give more inputs on many of the anti-stigma materials that were already developed but provided fewer novel ideas.

## CONCLUSION

The AEAG across both sites provided insight into contextually relevant issues that have been adopted into various anti-stigma resources developed for the ARTEMIS trial. They also critically appraised all the anti-stigma resources.

One suggestion from the AEAG members was to use social media for sharing information on mental health. However, given the study design and the risk of contamination this was not integrated in the trial though future projects are exploring better ways to adopt social media. Another suggestion of the adolescents was to have mental health awareness programmes for teachers which due to COVID related school closures and restrictions could not be developed appropriately and was not integrated into the final set of campaign strategies.

The setting up of AEAGs was a step in the direction of ethical research that views adolescents as active participants who are highly competent to design and deliver an intervention that directly affects them. The research team drew inspiration from the “postmodern pattern of sensemaking” [13], which visualises social communication as transparent and open-ended involving “the negotiation and renegotiation of meanings”. The researchers did not visualise co-creation simply as a research technique which used the creativity of the adolescents to value-add; instead, co-creation was viewed as a process that enabled participation from a position of equality rather than dominance, thus fostering democratisation [14], while creating meaning for both the researchers and the AEAG. Using the imagery of ARTEMIS as a ship to trace the Theory of Change for this project [12], the researchers were able to garner the attention of the AEAGs; in fact the researchers consider the AEAG as navigators who steer the ARTEMIS ship, avoiding “icebergs” (significant risks) and harnessing “winds”(facilitating factors) to ultimately reach the safety of the harbour (a state where adolescents are able to deal with tension, anxiety and other mental health issues). The experiences of co-creating with principal stakeholders (adolescents in the case of ARTEMIS) has important lessons for conducting community centred research.
